# Late-Onset Posttransplant Lymphoproliferative Disorders after Solid Organ Transplantation in Adults: A Case Series and Review of the Literature

**DOI:** 10.1155/2020/8247308

**Published:** 2020-02-10

**Authors:** S. Gandhi, E. Behling, D. Behrens, A. Ferber, R. Schwarting, T. Budak-Alpdogan

**Affiliations:** ^1^MD Anderson Cancer Center at Cooper, Camden, NJ, USA; ^2^Department of Pathology, Cooper University Hospital, Camden, NJ, USA

## Abstract

The posttransplant lymphoproliferative disorders (PTLDs) are a heterogeneous group of neoplasms that have wide variety of clinical and histological presentations. The management of PTLDs is challenging due to variety of involvement sites and histological types. The length and type of immunosuppression are correlated with the emergence of PTLDs, and most of the cases appear within the first two years after transplant. This case series describes five late-onset PTLDs with rare histological features and multiorgan involvement.

## 1. Introduction

Posttransplant lymphoproliferative disorders (PTLDs) comprise a heterogeneous group of lymphoid or plasmacytic proliferations/neoplasms, exhibit a wide spectrum of morphologies, and can occur after solid organ transplantation (SOT) or hematopoietic stem cell transplantation (HSCT).

While PTLD can develop at any time after organ transplantation, almost 60-80% of patients are diagnosed with PTLD within the first year after transplant [1, 2]. The incidence of PTLD again rises 4-5 years after SOT, and the mechanisms involving clonal proliferation in late PTLDs are obscure. Late-onset PTLD occurs within 2-10 years after SOT [1, 3, 4] and very late-onset PTLD occurs more than 10 years after transplantation [5].

The World Health Organization (WHO) divides PTLD into four major categories: nondestructive PTLDs (formerly known as early lesions), polymorphic PTLDs, monomorphic PTLDs, and classical Hodgkin lymphoma PTLDs [6]. Nondestructive PTLDs are characterized by architectural preservation of the involved tissue, usually with the formation of a mass lesion and/or significant Epstein-Barr virus (EBV) positivity, and may resemble florid follicular hyperplasia, plasmacytic hyperplasia, or infectious mononucleosis [6]. Polymorphic PTLDs, on the other hand, are characterized by heterogeneous infiltrates of small-, medium-, and large-sized lymphoid cells and plasma cells which efface or destroy the normal architecture of the involved tissue and do not fulfil the criteria for any of the recognized type of lymphomas described in immunocompetent patients [6]. In contrast, monomorphic PTLDs fulfil the criteria of one of the B cell or T/NK cell neoplasms that are recognized in immunocompetent patients and comprise the majority of PTLDs. Finally, there is also classical Hodgkin lymphoma PTLD which is almost always EBV-positive and fulfils the diagnostic criteria for classical Hodgkin lymphoma. Most PTLDs are B cell processes, with only 5-10% of PTLDs being T/NK cell or classical Hodgkin lymphoma type [6].

In this paper, we describe five cases of adult late-onset PTLD with emphasis on epidemiology, pathogenesis, unique presentations, and clinical implications within the scope of late-onset PTLD and its management.

## 2. Case Series

**Figure 1:**
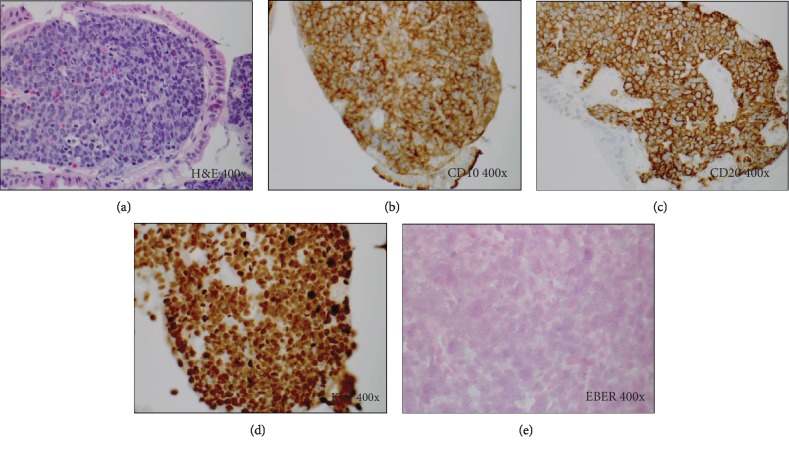
Histology and special studies from the duodenal lesion in Case 1. (a) H&E stain (400x). Duodenal infiltrate composed of sheets of large monomorphic lymphoid cells exhibiting a classic “starry sky” appearance, characteristic of Burkitt lymphoma. (b, c) Immunohistochemical stains (400x) for CD10 (b) and CD20 (c) show strong diffuse positivity, while immunostains for BCL2 and TdT (not shown) are negative. (d) Ki67 immunostain (400x) demonstrates a very high proliferation rate of >90%. (e) In situ hybridization (400x) for Epstein-Barr encoded RNA (EBER) is negative.

A 66-year-old male presented with abdominal pain. His past medical history was remarkable for chronic kidney disease, status post live donor renal transplantation 12 years prior. He was on tacrolimus and mycophenolate mofetil for immunosuppression. On evaluation, imaging was significant for a six-centimeter mass in the left upper abdomen involving the duodenum. Biopsy of the duodenal mass showed Burkitt-type PTLD, positive for MYC rearrangement in 95% of involved cells (Figures 1(a)–1(e)). In situ hybridization for EBV on the tumor specimen was negative (Figure 1(e)), and the patient's serum quantitative EBV titer with polymerase chain reaction (PCR) was negative. His overall diagnosis was consistent with stage IE, EBV-negative Burkitt-type PTLD.

He was treated with reduction of immunosuppression and rituximab, etoposide, prednisone, vincristine, cyclophosphamide, and doxorubicin (R-EPOCH) with intrathecal prophylaxis and growth factor support. He completed a total of six cycles of R-EPOCH. Upon completion of the chemotherapy, positron emission tomography (PET) scan showed complete resolution of the uptake in the site of disease, Deauville score 2. He continues to be in remission 19 months after completion of chemotherapy.

To our knowledge, this is the first reported case of very late-onset, EBV-negative, Burkitt-type PTLD in an adult.

## 3. Case 2

A 52-year-old male presented with a four-month history of malaise and 20-pound weight loss. His past medical history was significant for deceased donor kidney transplantation seven years prior to presentation and he was on immunosuppression. His imaging studies were remarkable for extensive retroperitoneal lymphadenopathy, multifocal intussusceptions in the lower pelvis, and a questionable sigmoidal apple core lesion. While his colonoscopy was unremarkable, esophagogastroduodenoscopy (EGD) was significant for a single ulcer in the body of the stomach. Pathology of this ulcer showed CD20-positive, polymorphous PTLD, and EBV testing was positive both by fluorescence in situ hybridization (FISH) for Epstein-Barr encoded RNAS (EBER) and immunohistochemistry for latent membrane protein.

He was diagnosed with stage IVB polymorphic-type PTLD. He was treated with four cycles of rituximab and reduction of immunosuppression. Following treatment, he had restaging PET-CT which showed increased metabolic activity of lymph nodes in the neck, chest, and abdomen as well as significant bone involvement.

**Figure 2:**
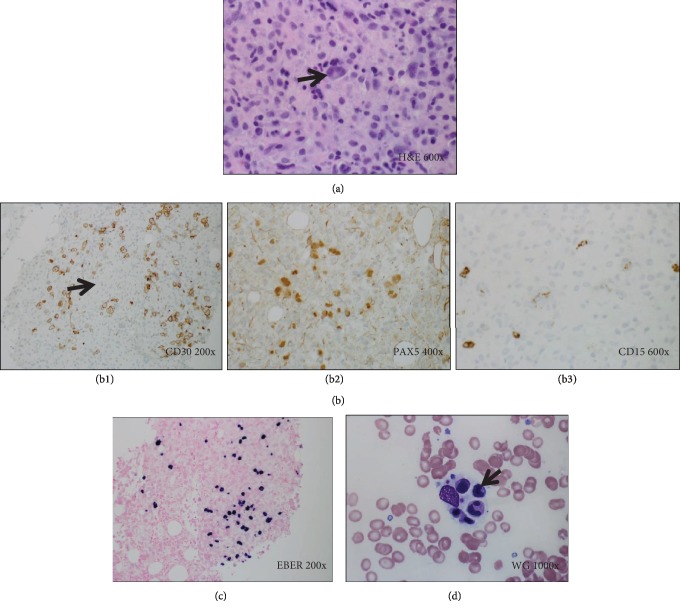
Histology and special studies from lymph node and bone marrow in Case 2. (a) H&E stain (600x). Bone marrow core biopsy showing classic Reed-Sternberg cells (arrow) within a mixed inflammatory background. (b) (1-3) Immunohistochemical stains demonstrate scattered large atypical cells which are positive for CD30 (b1), PAX5—moderate (b2), and CD15—subset weak (b3), but negative for CD45, CD20, and OCT2 (not shown). (c) EBER in situ hybridization (200x) is positive in the large atypical cells. (d) Wright-Giemsa stained bone marrow aspirate smear (1000x) showing conspicuous hemophagocytosis (arrow).

He remained pancytopenic and was readmitted to the hospital with fever and constitutional symptoms. He underwent extensive evaluation for infectious etiologies and was treated with empiric broad-spectrum antibiotics. Repeat bone marrow biopsy revealed effacement of the marrow space with atypical histiocytic and noncaseating granulomas, admixed with large and atypical multinucleated giant cells, compatible with classical Hodgkin-type PTLD (Figures 2(a)–2(c)). In situ hybridization was positive for EBER and negative for acid fast bacilli, fungal infection, and Bartonella species.

Liver biopsy was also performed and showed hepatic involvement of classical Hodgkin-type PTLD.

Due to severe hyperbilirubinemia, he was treated with gemcitabine and oxaliplatin. Rituximab was added given scattered CD20 positivity. He showed remarkable clinical response after three cycles; EBV quantitative PCR decreased from 16,669 copies/mL at diagnosis to undetectable. However, after his seventh cycle of chemotherapy, he was readmitted for persistent high fevers. Workup for infectious etiologies was again negative. Repeat bone marrow biopsy showed persistent Hodgkin-type PTLD and evidence of hemophagocytosis (Figure 2(d)). His therapy was changed to adriamycin, vincristine, and dacarbazine (AVD). Following two cycles of AVD, his PET-CT showed no evidence of disease. His care was then transitioned to another institution.

To our knowledge, this is the first case of adult and late-onset polymorphic PTLD with Hodgkin's transformation, complicated by hemophagocytic lymphohistiocytosis.

## 4. Case 3

A 52-year-old female with history significant for failed deceased donor renal transplantation followed by live renal transplantation 16 years prior, on chronic immunosuppression with prednisone, mycophenolate mofetil, and cyclosporine, presented with complaints of an enlarging lesion of the hard palate, cough, and fevers.

She underwent biopsy of this oral lesion. Pathology was consistent with a lymphoproliferative disorder comprised of large and atypical-appearing B lymphocytes, with positivity for CD20, PAX5, BCL6, focal CD10, MUM1, BCL2, and strong, diffuse positivity for CD30. There were no characteristic Reed-Sternberg cells present. In situ hybridization for EBV was positive in both small and large cells. The biopsy from the oral lesion considered as polymorphous PTLD.

**Figure 3:**
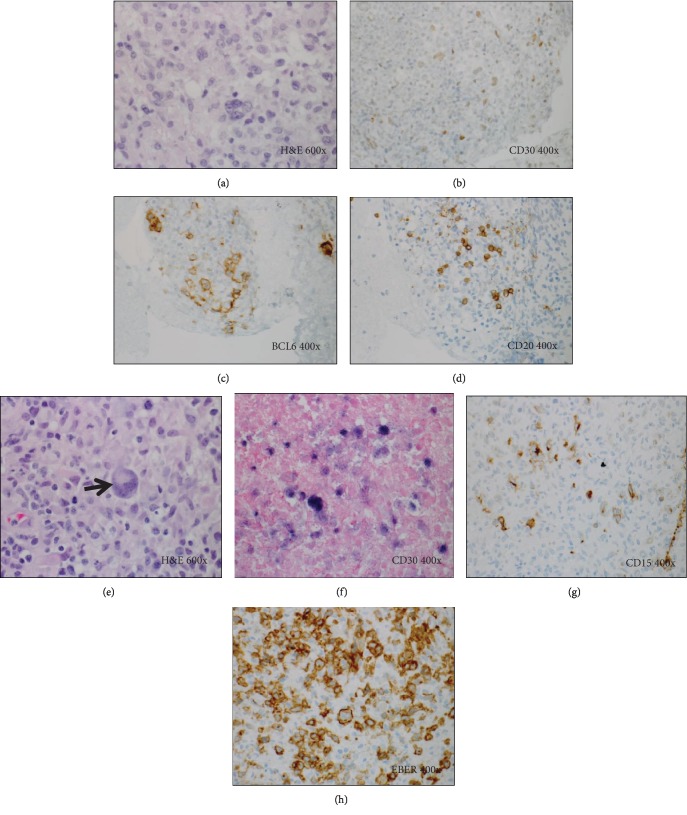
Histology and special studies from the left lung transbronchial biopsy (a–d) and brain lesion (e–h) in Case 3. (a) H&E stain (600x). Left lung transbronchial biopsy showing an atypical polymorphous lymphoid infiltrate containing scattered large Hodgkin/Reed-Sternberg cells within a mixed inflammatory background. (b–d) Immunohistochemical stains (400x) on the lung lesion show that the scattered large atypical cells are positive for CD30 (b), positive for BCL6 (c), focally positive for CD20 (d), but negative for CD45 (not shown). The tumor cells are also positive for EBER by in situ hybridization (not shown). (e) H&E stain (600x). Brain lesion also showing classic Reed-Sternberg cells (arrow). (f–h) Immunohistochemical and in situ hybridization studies (400x) on the brain lesion also show large tumor cells positive for CD30 (f), CD15 (subset) (g), and EBER (h).

Her CT chest was significant for masses at the left upper and left lower lobes adjacent to the left hilum, with multiple small nodular and infiltrative lesions in the right lung fields. Transbronchial biopsy revealed scattered large atypical cells with features of classic Reed-Sternberg cell. The cells were positive for CD30, BCL6 (weak), PAX5, and EBER by in situ hybridization (Figures 3(a)–3(d)). The tumor cells had variable and weak CD20 labeling. Cytology was consistent with EBV-positive PTLD with features of classical-type Hodgkin's lymphoma.

Reduction of the immunosuppression for one month decreased the size of the left lower lobe mass. However, she continued to have fever with elevated EBV titers and was therefore started on weekly rituximab at a dose of 375 mg/m^2^. She presented to the hospital after her first cycle with complaints of ataxia and headache. Magnetic resonance imaging (MRI) of the brain was significant for multiple regions of abnormal parenchymal and leptomeningeal enhancement with vasogenic edema in both supratentorial and infratentorial distribution. She underwent biopsy of a lesion which was consistent with involvement by EBV-positive posttransplant lymphoproliferative disorder with classical Hodgkin lymphoma features similar to the lung biopsy, positive for PAX5, CD30, scattered subset reactive to CD15, and minimal, weak CD20 reactivity (Figures 3(e)–3(h)). She received high-dose methotrexate at 3.5 g/m^2^ and rituximab, and restaging scans after four cycles were significant for interval progression.

Her therapy was changed to brentuximab and high cytosine arabinoside. Unfortunately, she developed left-sided weakness and drowsiness after her first cycle, and she was noted to have acute parenchymal hemorrhage with midline shift, likely secondary to necrosis of lymphoma lesion. She died secondary to complications from intraparenchymal hemorrhage. She survived nine months from the diagnosis of Hodgkin-type PTLD.

This case, to our knowledge, is the first in which a patient had two coexisting histologies of polymorphic PTLD and PTLD with features of classical-type Hodgkin lymphoma involving both the lung and CNS.

## 5. Case 4

A 73-year-old male presented five days after a fall, and initial CT scan of the head was significant for an abnormality in the right temporal region. His past medical history was notable for renal transplant 11 years prior to presentation; he was on active immunosuppression with mycophenolate mofetil and prednisone. His follow-up MRI of the brain showed a right medial temporal lobe lesion measuring 2.8 cm, suspicious for neoplasm, as well as two smaller lesions in the periventricular white matter. He underwent image-guided craniotomy for biopsy of the temporal lobe lesion. Pathology was consistent with an EBV-positive (EBER-positive in approximately 40% of cells), CD20, CD19, CD45, and MUM1-positive large cell lymphoma with extensive necrosis.

Unfortunately, this patient declined follow-up for his CNS-involving PTLD.

## 6. Case 5

A 74-year-old woman with history significant for live donor renal transplantation 10 years prior to presentation, on immunosuppression with tacrolimus, prednisone, and mycophenolate mofetil, presented to the hospital with four weeks of progressive right-sided weakness and daily frontal headaches. MRI of the brain demonstrated a lesion in the left posterior frontal lobe, concerning for metastasis. Her CT of the chest, abdomen, and pelvis did not reveal a primary malignancy.

**Figure 4:**
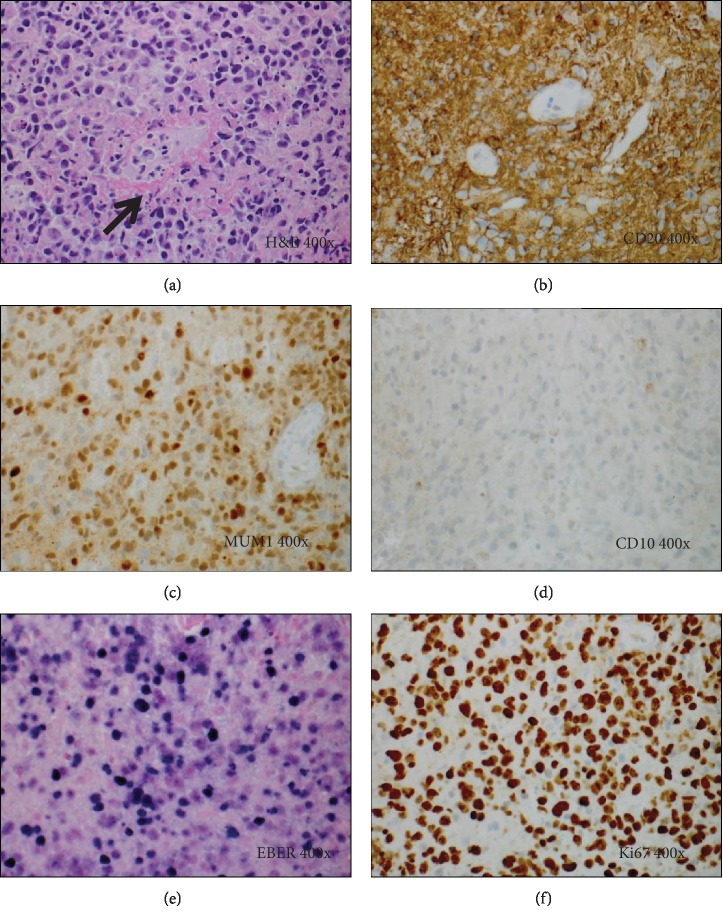
Histology and special studies from the brain lesion in Case 5. (a) H&E stain (400x). Brain lesion showing an atypical lymphoid infiltrate composed of sheets of large lymphoid cells with abundant foci of single cell necrosis/apoptosis, often in a perivascular distribution (arrow). (b–d) Immunohistochemical stains (400x) demonstrate that the lymphoid infiltrate is composed of sheets of large B cells which are positive for CD20 (b), MUM1 (c), and BCL2 (not shown), but negative for CD10 (d). (e) The tumor cells are positive for EBER (400x) by in situ hybridization. (f) Ki67 immunostain shows a high proliferative rate of 90%.

She underwent image-guided biopsy from the left posterior frontal mass. Pathology showed a nongerminal center-type, EBV-positive diffuse large B cell lymphoma with Ki67 of approximately 90%, consistent with a high-grade monomorphic PTLD. FISH studies for BCL2, BCL6, and MYC rearrangements were negative (Figures 4(a)–4(f)).

Rituximab was considered for treatment, as her poor performance status would not allow for treatment with standard regimens for primary CNS lymphoma. The patient and her family declined aggressive therapy.

## 7. Discussion

Our series represent a group of patient survivors of solid organ transplant who presented with late-onset PTLDs (Table 1). All, except one, had EBV-associated PTLDs assessed by EBER in situ hybridization. All of our patients presented with solid organ involvement: lung, CNS, liver, and gastrointestinal tract. The histological types were Burkitt lymphoma, diffuse large B cell lymphoma, and Hodgkin-type PTLD. Though all individual cases were unique in their presentation, our series reflected the heterogenous presentation of late PTLDs that was described in other published case reports and series [1, 5, 7].

PTLDs comprise approximately 20% of all malignancies after solid organ transplantation [8]. The incidence of PTLD in adults is dependent on several factors: the type of allograft, EBV infection, and degree of immunosuppression. The incidences of PTLD in renal and hepatic transplant recipients, thoracic transplant recipients, and small bowel transplant recipients are approximately 1-3%, 1.2-7.5%, and 20%, respectively [9, 10]. EBV plays an important role in the pathogenesis of PTLD, as many cases of PTLD are related to EBV primary infection or reactivation. Immunosuppression for prevention of transplant rejection results in a decrease in cytotoxic T lymphocyte activity, thus allowing for EBV DNA replication and the expression of virally mediated oncogenes in infected B cells [8]. EBV has been found to be positive in estimated 60% to 80% of cases of PTLD, most of which are early-onset. However, EBV-negative PTLD is now emerging as a distinct clinical entity [11].

Interestingly, recent studies have proposed that early- and late-onset PTLD affect different populations, have different morphologic characteristics, and have different behaviors [2, 3, 5]. Because our cases are examples of late-onset PTLD, we will focus on this subgroup.

### 7.1. EBV Status in Early-Onset and Late-Onset PTLD

Across several retrospective analyses, it has been observed that the presence of the EBV genome in the tumor is more prevalent in early-onset PTLD as compared to late-onset PTLD, and the relative proportion of EBV-negative lesions may increase over time after transplant [12]. Nelson et al. reported that EBV-negative PTLD occurred at a median of 50 months of posttransplantation, while EBV-positive cases of PTLD occurred within the first 10 months of transplantation [13]. In a retrospective series, Leblond et al. compared characteristics of EBV-negative and EBV-positive cases of PTLD. Approximately 84% of PTLD cases that were diagnosed within 2 years of SOT were EBV-positive, whereas 38% of PTLD cases diagnosed after 2 years of SOT were EBV-positive [14]. This observation is partly explained by the findings that median primary EBV infection onset and EBV reactivation after SOTs are 6 weeks and 2-3 months, respectively [13]. However, recent studies have shown that initial detection of EBV viremia can occur as late as a mean of 276 days [15].

Interestingly, four out of our five cases of late-onset PTLD were EBV-positive, so negative EBV status does not rule out possible PTLD in transplant patients. Series of early-onset PTLDs report better survival than what we observed in our series. To our knowledge, there have not been studies comparing the characteristics and behavior of EBV-negative and EBV-positive late-onset PTLD, possibly because of the rarity of the disease.

### 7.2. Localization of Early-Onset and Late-Onset PTLD

Previous evidence has shown that early-onset PTLD is more likely to occur within the allograft, whereas late-onset PTLD is commonly extranodal. Caillard's observations made further links between the location of PTLD and time from SOT. In this prospective study on PTLDs diagnosed during a 10-year period in a population of adult kidney recipients, it was noted that graft PTLD, central nervous system (CNS) PTLD, and gastrointestinal (GI) PTLD occurred within 2 years, between 2 and 7 years and between 6 and 10 years after SOTs, respectively [16].

Perhaps the most interesting finding in our PTLD case series was that two of our five patients had primary CNS PTLD, an observation which is quite rare. In fact, within the largest postmortem analysis of posttransplant recipients, only 2-7% of primary CNS PTLD were observed [17, 18].

Castellano-Sanchez et al. conducted a retrospective analysis of 12 cases of CNS PTLD. The latency period between transplantation and development of CNS PTLDs was a mean duration of 31 months from SOT [19]. Ten out of the 12 patients (83%) included in the study developed primary CNS PTLD within 37 months of SOT, one patient developed primary CNS PTLD 6 years after SOT, and one patient developed primary CNS PTLD 10.9 years after SOT. Aside from two patients, the majority of primary CNS PTLDs in this series were multifocal and involved different areas of the CNS (16). Finally, all but one case had EBV positivity. The histopathology of all 12 cases was consistent with monomorphic PTLD with features similar to diffuse large B cell lymphomas of the CNS in patients with HIV [19]. In another case series of ten patients with primary CNS PTLD, the average latency period for the development of PTLD after SOT was 5.59 years, with a range of 1.8 to 11.4 years [20]. Also within this case series, there were five cases of monomorphic, diffuse large B cell subtype PTLD, four cases of polymorphic PTLD, and one case of monomorphic, peripheral T cell PTLD of the CNS [20].

The presence of CNS involvement by PTLD, either isolated or in conjunction with other organ involvement, portends a poor prognosis. Penn and Porat reported that approximately 25% (289 of 1132) of allograft recipients who developed lymphomas had CNS involvement, most frequently in the brain, and in spite of treatment, 63% succumbed to their lymphoma [21].

### 7.3. Hodgkin Lymphoma PTLD (HL-PTLD)

Two of our five cases had classical Hodgkin-type PTLD, which is a rare histology in PTLD's. In one study investigating the epidemiology of PTLD in renal transplant recipients, six of 80 PTLD cases were Hodgkin-type; the majority of which were late-onset and EBV-positive [22]. Rosenberg et al. conducted a study to determine the differences between HL-PTLD and immunocompetent HL. Interestingly, a 5-year overall survival for HL-PTLD was significantly lower than immunocompetent HL (57% versus 80%, respectively), suggesting that despite histology, the two entities exhibit distinct biologic behavior [23].

### 7.4. Behavior of Early-Onset and Late-Onset PTLDs

There has been conflicting evidence in regard to the behavior, response to treatment, and overall survival in early-onset versus late-onset PTLD. The vast majority of studies have shown that early-onset PTLD portends a better prognosis, better response rate to reduction of immunosuppression, and improved overall survival compared to late-onset PTLDs [2, 7, 14]. Michonneau et al. reviewed the clinical and biologic data of kidney transplant recipients who developed late-onset PTLD and compared long-term outcomes before and after the advent of rituximab. Prior to the use of rituximab, complete remission was achieved in only 38% of patients, whereas patients treated with rituximab had a complete remission rate of 87% [7].

In addition, most late-onset PTLDs require both reduction of immunosuppression and aggressive chemotherapy, making allograft rejection a significant problem [24].

## 8. Conclusion

Posttransplant lymphoproliferative disorders (PTLDs) represent catastrophic complications of SOT. Non-Hodgkin lymphomas are the most common malignancy to occur after SOT [25]. The absolute rarity of the disease has limited the development of rigorous clinical guidelines based on randomized prospective trials. Our case series highlight the high degree of heterogeneity in time of onset after transplant, clinical presentation, histology, and clinical behavior of this disease.

## Figures and Tables

**Table 1:** Characteristics of each case of PTLD described, including age at diagnosis, type of SOT, time from SOT to PTLD diagnosis, location of PTLD lesions, immunosuppression, histologic subtype, EBV status, treatment, failure of SOT, and patient status (alive or deceased).

Case	Age at diagnosis (years)	SOT	Time from SOT to PTLD diagnosis (years)	PTLD location	Immunosuppressive regimen	Histologic subtype	EBV status	Treatments	SOT failure	Patient status
1	66	Deceased donor kidney	12	Duodenum	Tacrolimus, mycophenolate mofetil	Burkitt	Negative	ROI, R-EPOCH	No	Alive
2	53	Live donor kidney	7	Stomach, retroperitoneal (RP) lymph nodes (LNs), bone marrow	Tacrolimus, prednisone	RP LN: polymorphic, bone marrow: Hodgkin	Positive	ROI, rituximab, R-GEMOX, AVD	Yes	Alive
3	52	Deceased donor kidney, live donor kidney	30, 16	CNS, oropharynx, lung	Cyclosporine, mycophenolate mofetil, prednisone	Oropharynx: polymorphic, CNS and lung: Hodgkin	Positive	ROI, rituximab, high-dose MTX, brentuximab, high-dose cytarabine	No	Deceased
4	73	Kidney	11	CNS	Mycophenolate mofetil, prednisone	Diffuse large B cell	Positive	Unknown	Unknown	Unknown
5	74	Live donor kidney	10	CNS	Tacrolimus, mycophenolate mofetil, prednisone	Diffuse large B cell	Positive	None	Unknown	Unknown

SOT: solid organ transplantation; PTLD: posttransplant lymphoproliferative disorder; CNS: central nervous system; ROI: reduction of immunosuppression; R-EPOCH: rituximab, etoposide, prednisone, vincristine, cyclophosphamide, adriamycin; R-GEMOX: rituximab, gemcitabine, oxaliplatin; AVD: adriamycin, vinblastine, dacarbazine.
